# Estrogen attenuates physical and psychological stress‐induced cognitive impairments in ovariectomized rats

**DOI:** 10.1002/brb3.2139

**Published:** 2021-04-03

**Authors:** Mina Khaleghi, Mohammad Amin Rajizadeh, Hamideh Bashiri, Kristi Anne Kohlmeier, Fatemeh Mohammadi, Mohammad Khaksari, Mohammad Shabani

**Affiliations:** ^1^ Department of Physiology and Pharmacology Kerman University of Medical Sciences Kerman Iran; ^2^ Department of Physiology and Pharmacology Kerman University of Medical Science, Kerman Iran and Sirjan School of Medical Sciences Sirjan Iran; ^3^ Department of Drug Design and Pharmacology Faculty of Health Sciences University of Copenhagen Copenhagen Denmark; ^4^ Neuroscience Research Center Neuropharmacology Institute Kerman University of Medical Sciences Kerman Iran; ^5^ Endocrinology and Metabolism Research Center Kerman University of Medical Sciences Kerman Iran

**Keywords:** anxiety‐like behavior, cognitive impairments, estrogen, OVX rats, stress

## Abstract

**Introduction:**

Women are more vulnerable to stress‐related disorders than men, which is counterintuitive as female sex hormones, especially estrogen, have been shown to be protective against stress disorders.

**Methods:**

In this study, we investigated whether two different models of stress act differently on ovariectomized (OVX) rats and the impact of estrogen on physical or psychological stress‐induced impairments in cognitive‐behaviors. Adult female Wistar rats at 21–22 weeks of age were utilized for this investigation. Sham and OVX rats were subjected to physical and psychological stress for 1 hr/day for 7 days, and cognitive performance was assessed using morris water maze (MWM) and passive avoidance (PA) tests. The open field and elevated plus maze tests (EPM) evaluated exploratory and anxiety‐like behaviors.

**Results:**

In sham and OVX rats, both physical and psychological stressors were associated with an increase in EPM‐determined anxiety‐like behavior. OVX rats exhibited decreased explorative behavior in comparison with nonstressed sham rats (*p* < .05). Both physical stress and psychological stress resulted in disrupted spatial cognition as assayed in the MWM (*p* < .05) and impaired learning and memory as determined by the PA test when the OVX and sham groups were compared with the nonstressed sham group. Estrogen increased explorative behavior, learning and memory (*p* < .05), and decreased anxiety‐like behavior compared with vehicle in OVX rats exposed to either type of stressor.

**Conclusions:**

When taken together, estrogen and both stressors had opposite effects on memory, anxiety, and PA performance in a rat model of menopause, which has important implications for potential protective effects of estrogen in postmenopausal women exposed to chronic stress.

## Graphical abstract

Findings from the current study delineated that exposure to both physical stress and psychological stress can cause cognitive disorders and increase anxiety‐like behaviors. These findings suggest that female rats are vulnerable to both stressor types, and effects impact motor and cognitive behaviors of the animals.

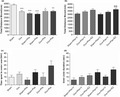

**Highlights**

- Two stress modalities exerted similar effects on exploration and anxiety.
- Swim stress impaired avoidance learning and increased anxiety‐like behaviors.
- Estrogen improved memory retrieval and reduced anxiety‐like behaviors.

## 1 INTRODUCTION

Stress is a double‐edged phenomenon which can be productive or counterproductive. While a quick response to demands or threats is promotive of survival, prolonged stress can cause counterproductive dyshomeostasis (Sapolsky, 1996). While very intense or long‐lasting stress can lead to an advantageous biological equilibrium termed eustress, for example as seen in the improved hemodynamic status of the heart due to exercise‐induced conditioning (Tafet & Bernardini, 2003), prolonged stress often leads to a maladaptive state (Selye, 1976) which can lead to injury or malady termed allostasis (McEwen, 1998). Stress can be divided into two categories: physical stress (e.g., swim stress, exercise, electric shocks) and psychological stress (e.g., Immobilization/restraint, unpredictable stress; García‐Bueno et al., 2008). Whether induced by physical or psychological stimuli, stress increases activation of the hypothalamic–pituitary–adrenal (HPA) axis, resulting in release of corticotropin‐releasing factor (CRF) and generation of glucocorticoids (GCs) from the adrenal gland, which freely cross the blood–brain barrier and interact with two different receptor types: the mineralocorticoid receptor (MR or Type I) and the GC receptor (GR or Type II), which are found in the brain (Kadmiel & Cidlowski, 2013). When the GR is activated by GCs, this can lead to potentially long‐lasting effects on neural performance that is controlled by the nuclei where these receptors are located (Saleh et al., 2017). While GC receptors are distributed throughout the brain, they are abundant in the hippocampus which affects specified modalities of cognition, the amygdala which affects fear responses and anxiety, and the prefrontal cortex that governs working memory and extinction of learning (Marin et al., 2019; Rajizadeh, Esmaeilpour, et al., 2020). Accordingly, chronic stress leading to long‐lived generation of GCs would be expected to affect all of the cognitive behaviors controlled by the hippocampus, amygdala, and the prefrontal cortex (McEwen, 2012). Consistent with this, exposure to stress or treatment with corticosteroids leads to impaired spatial memory, likely due to actions on GR in the hippocampus (Goodman et al., 2015).

Women have been shown to be more vulnerable to stress‐induced cognitive disorders than men (Handa & Weiser, 2014), with anxiety as the most prevalent cognitive disorder exhibiting a sex‐difference (Kessler et al., 2009). Women are at about a two‐fold higher risk for anxiety disorders compared with men, and sex hormones have been suggested to play a role in the prevalence rate between the sexes (Li & Graham, 2017). Consistent with this, depression and anxiety behaviors have been shown during menstruation, the postpartum period and ovarian resection that are all conditions with fluctuating levels of estrogen and antidepressant and anxiolytic actions have been elicited in estrogen‐treated women (Pinkerton et al., 2010). Bilateral ovarian resection, which is a model of surgical menopause has an effect on depression‐like behaviors in rodents (Xu et al., 2019). Using this model in mice, low estrogen plasma levels have been associated with anxiety and depression‐like behaviors, and estrogen administration has reduced symptoms of these disorders (Lagunas et al., 2010). Further, estrogen has been shown to have effects on brain structures involved in cognitive‐based behaviors (Ghazvini et al., 2016; Sheppard et al., 2019). Although there are many investigations of the beneficial roles of estrogen on stress‐induced complications (Dumas et al., 2012; Hokenson et al., 2021), there are few studies regarding its behavioral effects on physical or psychological stress in laboratory animals. Due to the prevalence of stress among women, especially postmenopausal women, and its destructive effects on cognition, it is necessary to find a way to reduce stress' negative effects. Benefits of estrogen on stress‐associated outcomes have not been well studied. Accordingly, in this study, we examined the effects of estrogen on disorders of cognition and anxiety‐like behavior in models of physical and psychological stress in healthy and ovariectomized female rats.

## 2 MATERIALS AND METHODS

### 2.1 Animals

Adult female Wistar rats at 21–22 weeks of age (weighting 200–250 g) were utilized for this investigation. Animals were housed under a 12‐hr light–dark cycle (lights on: 07:00–19:00 hr) with temperature control (23 ± 1°C) and full access to food and water. Animals were kept under standard conditions (Ethics code: 96000603) optimized to minimize pain and discomfort according to the guidelines adhered to by Kerman University of Medical Sciences, Kerman, Iran, which included group housing.

### 2.2 Experimental procedure

Ninety‐six female rats (Figure S1) were either ovariectomized (OVX) or subjected to sham surgery (sham) and allocated into twelve groups (*n* = 8 in each group). Two groups were not exposed to stress (OVX and Sham), whereas five groups were exposed to physical stress (Phs), and the remaining five groups were exposed to psychological stress (Pss), and in some cases, the rat was also exposed to 17 β‐estradiol (E2; Purchased from Sigma company, Cas number: 50‐28‐2) solubilized in sesame oil or only to the sesame oil vehicle. E2 injections 10 μg/kg, s.c. were conducted on the first and third days of stress (Pandaranandaka et al., 2009).

### 2.3 Surgical process

Reciprocal ovariectomy was done under general anesthesia (60 mg/kg ketamine and 10 mg/kg xylazine). Ovaries were extracted through a mid‐abdominal cut. All of the ovariectomized rats were utilized 2 weeks after of surgery to prevent or minimize any hormonal affect originating from their reproductive cycle (Rajizadeh, Sheibani, et al., 2020). For the sham group, just a mid‐abdominal cut was made, and ovaries were not removed.

### 2.4 Stress exposures

Rats were subjected to either a psychological or a physical stressor while housed in a glass aquarium (each house: 25∗30∗45 cm). The stress process was initiated at day 15 postsurgery and maintained for the subsequent 7 days. Physical stress consisted of the filling of the aquarium with water (20 ± 2°C) via an electric pump, which necessitated the rat to swim. After 5 min of swimming, the water was evacuated, and after 10 min, the house was again filled with water. This process alternated in this manner for 1 hr. Psychological stress was effectuated by witnessing of the groups undergoing swimming stress by the psychologically stressed groups (Nazeri et al., 2017). This resulted in 12 treatment groups. The time interval between the injections and the testing was equal in all animals. Each group went through four different behavioral studies, which were performed after stress procedures in the following order: open field test (OFT), elevated plus maze, morris water maze (MWM) and passive avoidance (PA) task (Behavioral procedures timeline, Figure S2).

### 2.5 Open Field Test (OFT)

This test was performed to assess the impact of physical or psychological stress on anxiety‐like behaviors and exploratory behavior (rearing and grooming). The apparatus consisted of a field made of checker‐boarded Plexiglass (90*90*45 [H] cm) with a surrounding wall of the field. Rats were placed in the center of the field, and their behavior was recorded by a video tracking system (Ethovision 7). Total distance moved (TDM), velocity, and the total time elapsed in center and surround were analyzed for each rat. At the end of each experiment, the animals were extracted from the box and the field was sterilized with a cloth containing ethanol 10%, allowed to dry, and thereafter, the next rat was placed in the field (Shabani et al., 2012).

### 2.6 Elevated Plus maze (EPM)

The EPM was utilized to evaluate the anxiety‐like behaviors of the animals. The wooden maze consisted of two closed arms and two open arms with a camera recording device (50∗50∗50 cm). Rats were placed in the center of the maze, and the number of entries into the closed and open arms, and time spent in each arm was recorded (Aghaei et al., 2015; Razavinasab et al., 2020).

### 2.7 Morris water maze (MWM)

The MWM was used to assess spatial learning and memory. The MWM consisted of a black circular pool (140 cm wide, 45 cm high) filled with water and surrounded by visual cues on the walls observable to the rats. Water temperature was set between 21°C and 23°C. A platform, either visible or submerged (15 cm wide and 35 cm height) was embedded 1.5 cm above or below the water surface. All the experiments were done between 8:00 a.m. and 12:00 p.m. Each rat was examined in three blocks of trials, and each block consisted of four trials begun from various locations in the MWM. The inter‐block interval was at least 30 min for each rat. Rats were placed in one of the quadrants of the water pool with their face directed toward the pool wall. The location of the platform did not change in the acquisition trial. Rats were monitored for 60 s during which the platform was found. When found, a 20–30 s break was given to the rat on the platform. The rat was returned to its cage, and again, after 20–30 s, the rat was placed into the water. The time spent and distance moved by the rats to find the platform were recorded by the software. To evaluate the spatial memory of the rats, a single probe trial was accomplished 2 hr after the last block of trials. In the probe trial, the platform was extracted from the tank and rats were permitted to swim for 60 s in the tank. Distance moved and time spent in the target quadrant were recorded for each rat as an index of spatial memory retention (Aghaei et al., 2015).

### 2.8 Passive avoidance (PA) test

The PA test was used to assess associative learning and memory in rats. Following training, an animal learns to eschew an environment in which a prior unpleasant stimulus had been delivered. Here, PA learning was evaluated using an inhibitory PA paradigm. In short, a shuttle box apparatus with dimensions of 100 [L] × 25 [W] × 25 [H] (cm), which included a light and dark chamber that were divided by a door was utilized. In the learning phase of the test, each rat was habituated to the test device by placing the individual in the light chamber for 5 min before a return to the home cage. The next day, the rat was placed into the light chamber, the door was opened, and the rat was permitted to move to the dark chamber. Following a closure of the door, an electric shock (0.5 mA, 2 sec) was given via wires embedded in the floor of the dark chamber. This final part of the process was repeated up to five times at 1 hr intervals until the rat learned to avoid the dark chamber and remained in the light chamber for at least 120 sec. The number of shocks needed for learning was recorded. The evaluation phase of the test was performed 24 hr after the learning phase. The animal was placed in the light chamber (door closed), and after 30 sec, the door opened and the time until the animal entered the dark chamber was recorded as the step‐through latency (STL). The total time elapsed in the dark chamber (time in the dark chamber, TDC) during a period of 5 min after door opening was also recorded (Aghaei et al., 2015).

### 2.9 Plasma corticosterone and estrogen measurement

At the end of all experiments, the influence of stress and estrogen on corticosterone levels was assessed in a subset of the individuals from all groups (*n* = 6 in each group). All subjects were euthanized between 12:00 a.m. and 12:30 a.m., which was 24 hr after the end of the MWM probe test. Rats were anesthetized with CO_2_. Promptly after decapitation, blood was gathered on ice in plastic polyethylene tubes containing Na^2^–EDTA as an anticoagulant. Blood was centrifuged at 2,600 rpm for 20 min at 4°C. The plasma was placed into micro tubes and refrigerated at −80°C and analyzed with ELISA kits intended to detect mouse and rat corticosterone and 17 β‐estradiol. Analysis was conducted by an investigator blinded to the treatment of the animals.

### 2.10 Statistical analysis

Corticosterone levels, as well as all data from the MWM probe trials, EPM, and PA tests were analyzed by a one‐way analysis of variance (ANOVA). The time spent and distance moved to find the hidden platform in the MWM training in the acquisition phase were analyzed utilizing a two‐way analysis of variance (ANOVA) along with repeated measures to assess differences in learning rates between groups (with group and blocks as factors). When statistical significance was found between the groups, a Tukey's post hoc multiple comparison test was applied to detect between which groups the significance was present. The data were expressed as means ± *SEM*, and *p* < .05 was considered statistically significant.

## 3 RESULTS

### 3.1 17 β‐estradiol (E2) attenuated stress‐induced explorative and anxiety‐like behaviors impairments in OVX rats

**FIGURE. 1:**
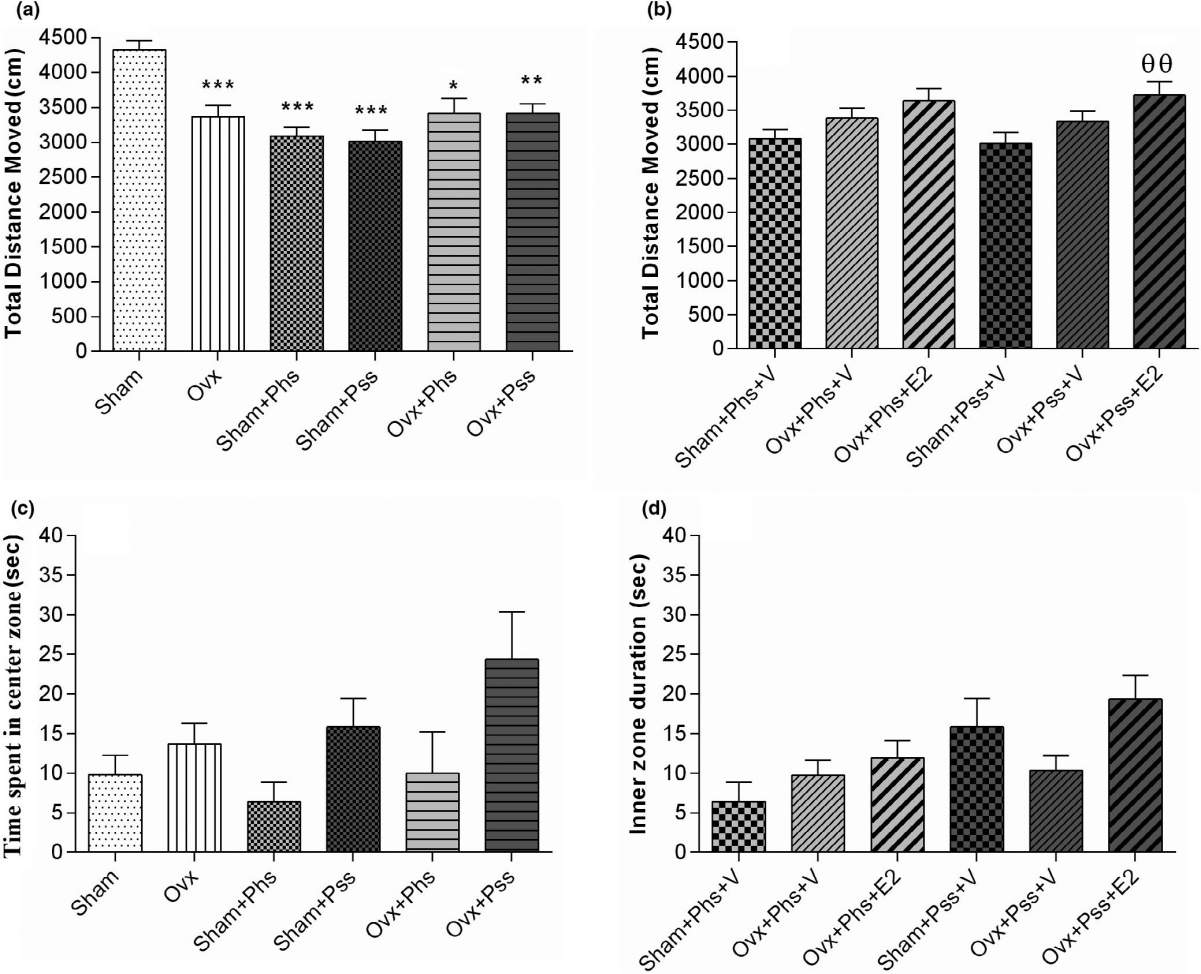
The effects of 17 β‐estradiol (E2), physical stress (Phs), and psychological stress (Pss) on anxiety‐like behavior and exploratory activity in the open field test. Total distance moved (a, b) and time spent in the inner zone (c, d) exhibited a significant difference among groups. Total distance moved in sham and ovariectomized (OVX) stress groups was lower than that seen in the sham group who did not experience stress (a) and E2‐treatment in the OVX + Pss group decreased the total distance traveled compared with OVX + Pss + Vehicle (V) group (b). There was no significant difference between the groups in the time spent in the center of the area (c, d). One‐way ANOVA was used for analysis of these data. Mean ± *SEM*. (*) *p* < .05 versus Sham. (**) *p* < .01 versus Sham. (***) *p* < .001 versus Sham. (θθ) *p* < .01 versus OVX + Pss + V

Anxiety‐like and exploratory behaviors were evaluated with the OFT. The total distance moved by the OVX group was lower than that of the sham group (Figure 1a, *p* < .001). Exposure to physical or psychological stressors was associated with a smaller total distance moved by both the sham and OVX groups than that seen in the sham group not exposed to stress (Figure 1a, *p* < .05). While no significant difference was noted in the physically stressed groups, when E2 was administered to the OVX group exposed to psychological stress, a significant increase in distance traveled was seen (Figure 1b, *p* < .01). There was no significant difference between any of the groups in the time spent in the center of the arena (Figure 1c) and exposure to E2 had no effect on this index (Figure 1d). These data suggest that estrogen has an effect on reducing deficits induced in exploratory behavior in OVX animals exposed to psychological stress.

### 3.2 17 β‐estradiol (E2) ameliorates stress‐induced anxiety‐like behaviors

**FIGURE. 2:**
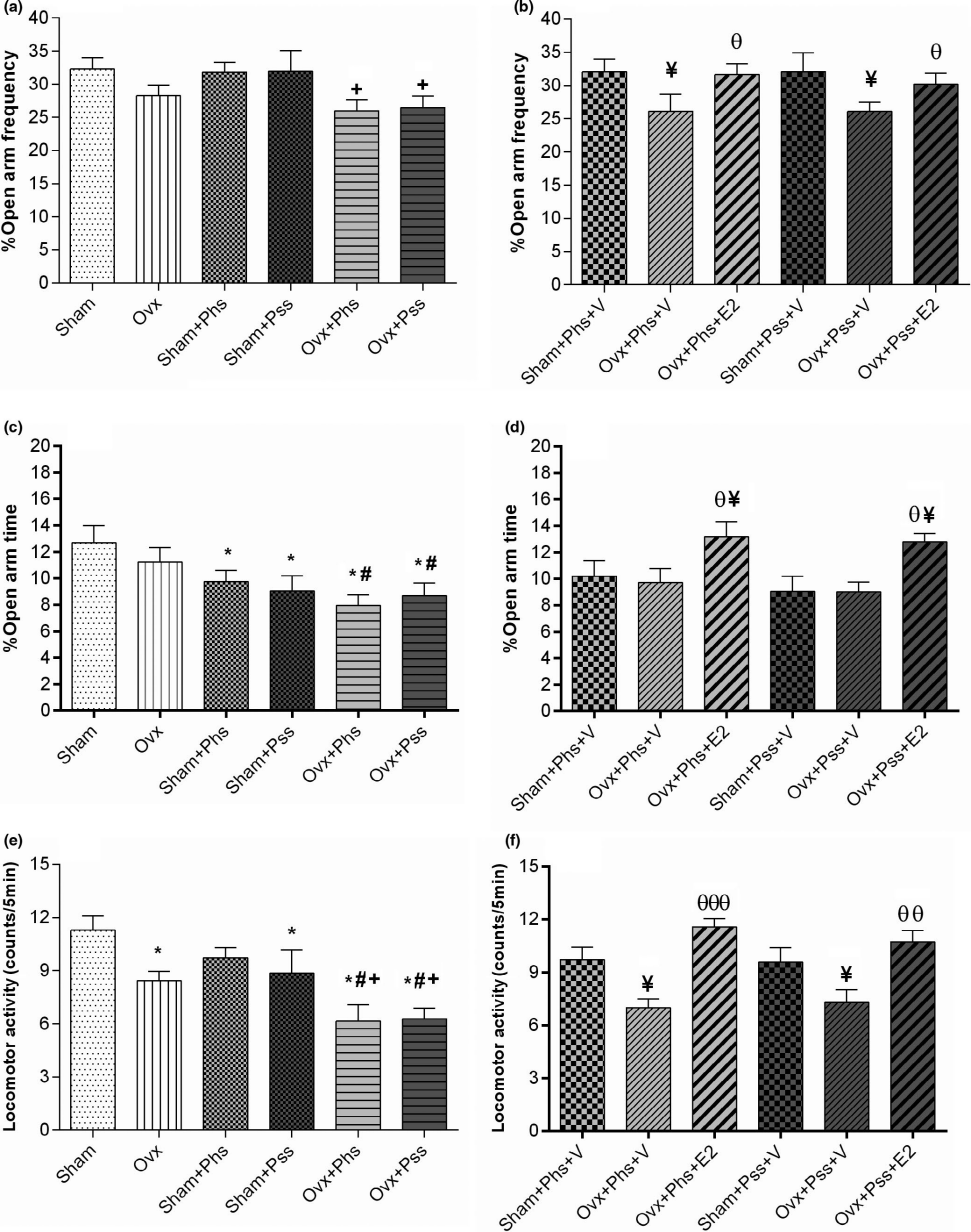
The effects of 17 β‐estradiol (E2), physical stress (Phs), and psychological stress (Pss) on anxiety‐like behavior in the Elevated plus maze. Both types of stress in OVX groups reduced the percentage of the frequency of time spent in the open arm compared with sham + Phs and sham + Pss groups (a). E2 in the OVX groups increased this index (b). Both types of stress in OVX groups decreased the percentage of time spent in open arms compared with sham groups (c) and E2 increased this parameter (d). Both modalities of stress decreased locomotor activity in the OVX groups (e), and E2 resulted in an increase in this parameter (f). One‐way ANOVA was used for analysis of these data. Mean ± *SEM*, ( + ) *p* < .05 versus Sham + Pss and Sham + Phs. (¥) *p* < .05 versus Sham + Phs + V and Sham + Pss + V. (θ) *p* < .05 versus OVX + Phs + V and OVX + Pss + V. (θθ) *p* < .01 versus OVX + Phs + V and OVX + Pss + V. (θθθ) *p* < .001 versus OVX + Phs + V and OVX + Pss + V. (*) *p* < .05 versus Sham. (#) *p* < .05 versus OVX

The effects of E2 on anxiety‐like behavior were further evaluated in the EPM. OVX rats did not show a difference when compared to sham animals, suggesting that ovariectomy does not heighten anxiety. A significantly reduced percentage of entries into the open arms was noted in the OVX group exposed to psychological and physical stressors when compared to the same parameter in sham rats exposed to the same stressors (Figure 2a, *p* < .05). Further, in vehicle‐treated animals, a smaller percentage of open arm entries was seen in OVX groups exposed to both modalities of stressor when compared to the sham group undergoing identical psychological and physical stressors (Figure 2b, *p* < .05). Treatment with E2 increased the number of entrances into the open arms in both OVX + Phs + E2 and OVX + Pss + E2 groups when compared to those seen in the corresponding vehicle groups (Figure 2b, *p* < .05). From these results, we conclude that estrogen has a positive effect in reducing stress‐induced, anxiety‐like behaviors in OVX rats.

No significant difference was seen in the percentage of time spent in the open arm between OVX and sham groups (Figure 2c). When exposed to physical or psychological stress, the sham and OVX groups exhibited a significantly reduced percentage of time in the open arm when compared to the sham group who did not experience stress (Figure 2c, *p* < .05), demonstrating that stress had similar effects on both OVX and sham groups.

A significantly lower percentage of time spent in the open arms was observed in both OVX groups exposed to the stressors when compared to this parameter in the nonstressed OVX group (Figure 2c, *p* < .05). Exposure to E2 in both the OVX groups exposed to stressors resulted in a significantly increased percentage of time spent in the open arm compared with the other groups (Figure 2d, *p* < .05). These data indicate that estrogen ameliorates the anxiogenic effect of psychological and physical stress as evaluated by the EPM. Locomotor activity in the OVX group was significantly reduced when compared to that in the sham group (Figure 2e, *p* < .05). In addition, in the OVX + Phs, the OVX + Pss and the sham + Pss groups, a significantly lower amount of locomotor activity was observed when compared to the nonstressed sham group, and there was no difference between the locomotor activity in the sham animals exposed to physical stress with that seen in the OVX animals, suggesting a lack of an additive effect (Figure 2e, *p* < .05). However, locomotor activity was significantly reduced in the OVX + Phs and OVX + Pss groups when compared to the OVX group suggesting an additive effect of loss of estrogen and stress‐mediated responses (Figure 2e, *p* < .05). In the OVX + Phs and OVX + Pss groups, a significant decrease in locomotor activity was observed compared with the respective sham groups exposed to the two stressors (Figure 2e, *p* < .05). Unexpectedly, OVX + Phs + V and OVX + Pss + V groups exhibited a significantly lower degree of locomotor activity when compared to sham + Phs + V and sham + Pss + V groups (Figure 2f, *p* < .05). Exposure to E2 increased locomotor activity significantly in both OVX groups exposed to stressors when compared to activity in the stress‐exposed OVX groups exposed to vehicle (Figure 2f, Phs: *p* < .01 and Pss: *p* < .001).

### 3.3 The influences of 17 β‐estradiol (E2), physical and psychological stress on spatial cognition in MWM task

**FIGURE. 3:**
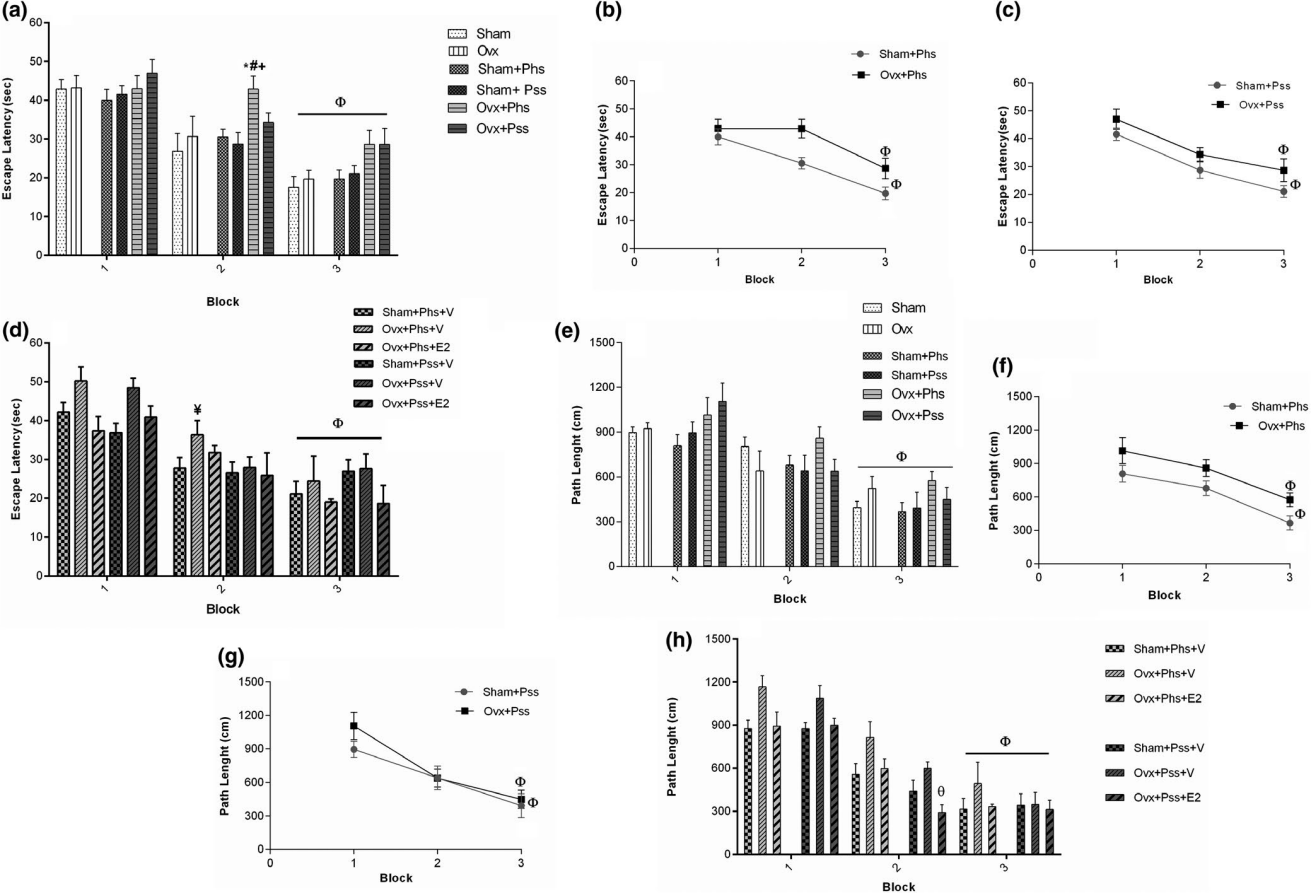
The effects of 17 β‐estradiol (E2), physical stress (Phs), and psychological stress (Pss) on spatial learning in the morris water maze. Phs increased escape latency in OVX + Phs group in block 2 compared with Sham, OVX and Sham + Phs group (a). Escape latency in block 3 significantly decreased compared with that seen in block 1 in all groups (a–c). Escape latency in OVX + Phs + V was higher than Sham + Phs + V in block 2 (d). In E2 groups in block 3, the escape latency was lower than that in block 1 (d) Path Length in block 3 was lower than that seen in block 1 (e–g). In E2 groups in block 3, the path length was lower than that recorded in block 1 (h). A two‐way, repeated measures ANOVA was used for analysis of these data. Mean ± *SEM*, (*) *p* < .05 versus Sham. (#) *p* < .05 versus OVX. (+) *p* < .05 versus Sham + Phs and Sham + Pss. (Ф) *p* < .05 versus Block 1. (¥) *p* < .05 versus Sham + Phs + V and Sham + Pss + V. (θ) *p* < .05 versus OVX + Phs + V and OVX + Pss + V

Physical stress in the OVX group was associated with a significant increase in block 2 of the escape latency defined as the time spent to find the hidden platform when compared to escape latency in the sham, the OVX and the sham + Phs groups, suggesting a compounding effect of ovariectomy (Figure 3a, *p* < .05). In block 3, within all groups a significantly lower escape latency was seen when compared to block 1, indicating an effect of training (Figure 3a, *p* < .05). In block 3, a significant decrease in escape latency was observed in the OVX rats (Phs: Figure 3b, *p* < .05; Pss: Figure 3c, *p* < .05) and the sham animals (Phs: Figure 3b, *p* < .05; Pss: Figure 3c, *p* < .05) exposed to stressors when compared to that seen in Block 1 in the same groups (Figure 3b,c).

**FIGURE. 4:**
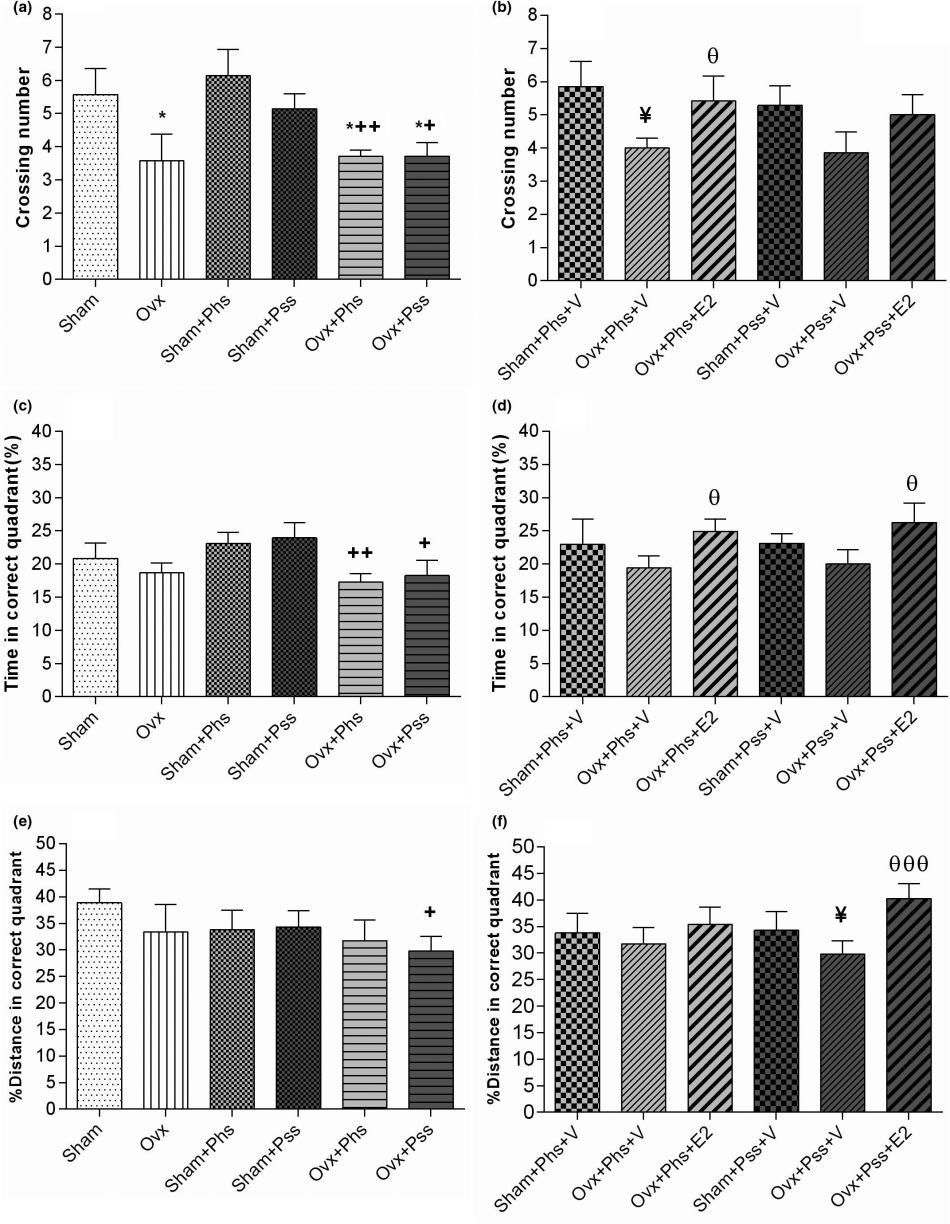
The effects of 17 β‐estradiol (E2), physical stress (Phs), and psychological stress (Pss) on spatial memory in morris water maze task. Ovariectomy and its combination with stress decreased crossing number (a) and E2 increased crossing number in OVX + Phs significantly (b). Combination of ovariectomy with both types of stress decreased time spent in the correct or target quadrant (c) and E2 in both situations improved this index (d). Only OVX + Pss resulted in a significant distance moved within the correct quadrant (e) and E2 significantly improved this measure (f). One‐way ANOVA was used for analysis of these data. Mean ± *SEM*, (*) *p* < .05 versus Sham. (+) *p* < .05 versus Sham + Phs & Sham + Pss. (++) *p* < .01 versus Sham + Phs and Sham + Pss. (¥) *p* < .05 versus Sham + Phs + V and Sham + Pss + V. (θ) *p* < .05 versus OVX + Phs + V and OVX + Pss + V. (θθθ) *p* < .001 versus OVX + Phs + V and OVX + Pss + V

In the OVX + Phs + V group, a significant increase in escape latency was seen in block 2 when compared to the sham + Phs + V group (Figure 3d, *p* < .05). A significantly reduced escape latency was seen in the estrogen‐treated groups between the sham and OVX rats exposed to physical and psychological stress (in block 3 compared with block 1; Figure 3d, *p* < .05). These results demonstrate that estrogen treatment improved learning impairments associated with the two types of stressors. No significant difference between sham and OVX groups in the three blocks was observed in path length, which was the distance moved to find the hidden platform (Figure 3e). In block 3, a significantly smaller path length was observed in all groups when compared to that seen in block 1 (Figure 3e, *p* < .05), which indicates that the learning process occurs in all groups. In block 3, the OVX group exposed to physical stress demonstrated a significantly higher path length when compared to the sham group exposed to a similar stress, suggesting a greater stress‐induced impairment in learning in ovariectomized rats (Figure 3f, *p* < .05). In block 3, a significant decrease in path length was observed in all groups when compared to path length in block 1 (Figure 3f,g, *p* < .05). In block 2, a significant effect of estrogen in decreasing the path length in OVX rats exposed to the psychiatric stressor was seen (Figure 3h, *p* < .05). The number of animal entrances into the target quadrant referred to as the crossing number was significantly lower in the OVX group, the OVX + Phs group, and the OVX + Pss group than the sham animals (Figure 4a, *p* < .05). This index was significantly lower in OVX + Phs and OVX + Pss groups when compared to that in the sham + Phs and sham + Pss groups, respectively (Figure 4a, Phs: *p* < .01 and Pss: *p* < .05). The crossing number was significantly lower in the OVX + Phs + V group when compared to that in the sham + Phs + V group (Figure 4b, *p* < .05); however, E2 in the OVX animals exposed to physical stress was able to reverse this index, which was a significant effect (Figure 4b, *p* < .05). The OVX + Phs and OVX + Pss showed a significantly decreased percentage of time in the correct quadrant when compared to the sham + Phs and sham + Pss groups (Figure 4c, *p* < .05 and *p* < .01, respectively). Estrogen treatment in both stress‐exposed OVX groups resulted in a significant increase in the percentage of time in the correct quadrant compared with vehicle‐treated, physical and psychologically‐stressed OVX rats (Figure 4d, *p* < .05).

In the groups exposed to psychological stress, there was a significantly lower distance traveled in the correct quadrant in the OVX group when compared to the sham group (Figure 4e, *p* < .05). Similarly, in the vehicle‐treated groups exposed to psychiatric stress, there was a significantly lower distance traveled in the correct quadrant in the OVX animals when compared to distance traveled in the sham group (Figure 4f, *p* < .05). Further, in the OVX group exposed to psychiatric stress, treatment with E2 significantly increased this index when compared to distance traveled in the OVX, psychiatric stress group treated with vehicle (Figure 4f, *p* < .01).

#### 3.3.1 Stress‐induced disturbances of function evaluated by the PA test can be partly alleviated by 17 β‐estradiol (E2)

**FIGURE. 5:**
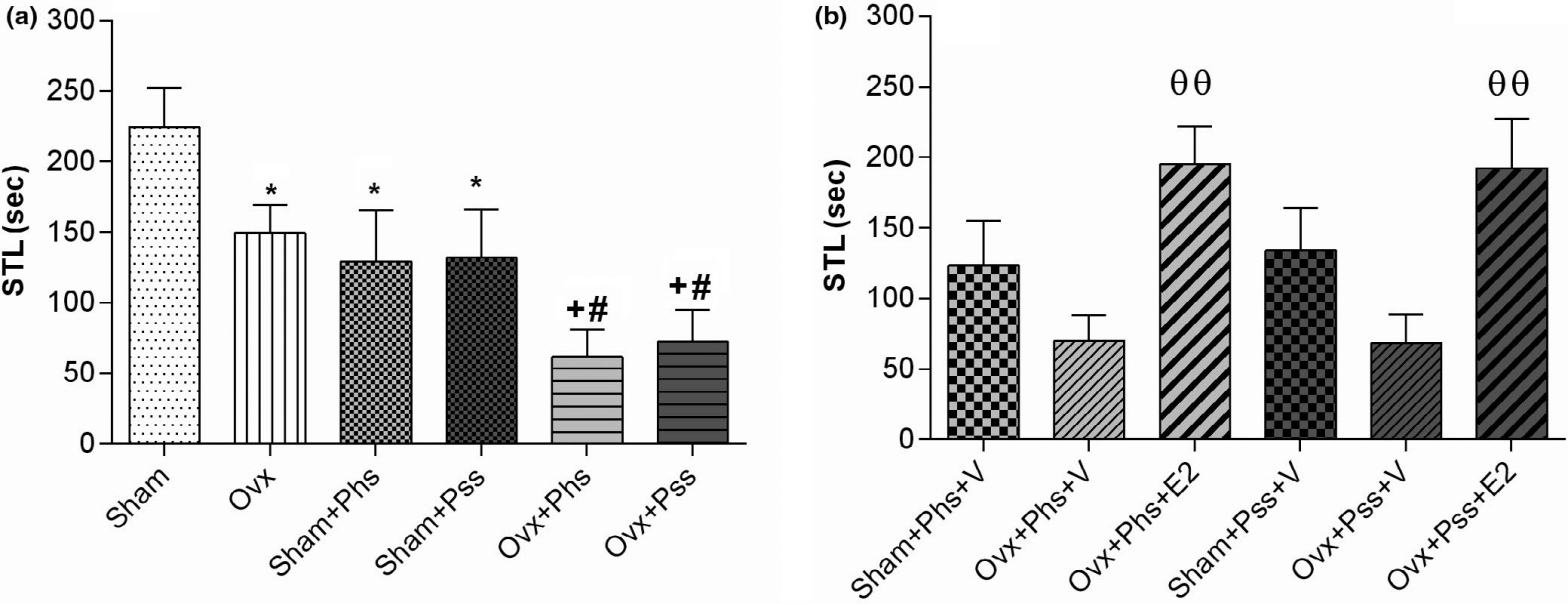
The effects of 17 β‐estradiol (E2), physical stress (Phs), and psychological stress (Pss) on passive avoidance memory in a shuttle box task. Ovariectomy and both types of stress decreased STL (a) and E2 ameliorated this parameter (b). One‐way ANOVA was used for analysis of these data. Mean ± *SEM*, (*) *p* < .05 versus Sham. (#) *p* < .05 versus OVX. (+) *p* < .05 versus Sham + Phs and Sham + Pss. (θθ) *p* < .01 versus OVX + Phs + V and OVX + Pss + V

When using the PA test, a significant decrease was seen in the STL of the first entry into the dark chamber in the OVX group when compared to the sham group (Figure 5a, *p* < .05). In addition, both stressors were associated with significantly lower STLs in sham animals when compared to those in the nonstressed groups (Figure 5a, *p* < .05). In OVX animals, both modalities of stress were associated with a significantly lower STL than in the nonstressed OVX group (Figure 5a, *p* < .05). Further, both modalities of stress caused a significantly lower STL in the OVX animals when compared to the STL seen in the sham animals when exposed to similar stressors (Figure 5a, *p* < .05). When OVX stressed groups were treated with estrogen, the STL was significantly increased when compared to vehicle‐treated, stressed OVX groups (Figure 5b, *p* < .01).

### 3.4 Plasma 17 β‐estradiol (E2) levels in sham, OVX and physical and psychological stress rats on 0, 15, and 21 days

**FIGURE. 6:**
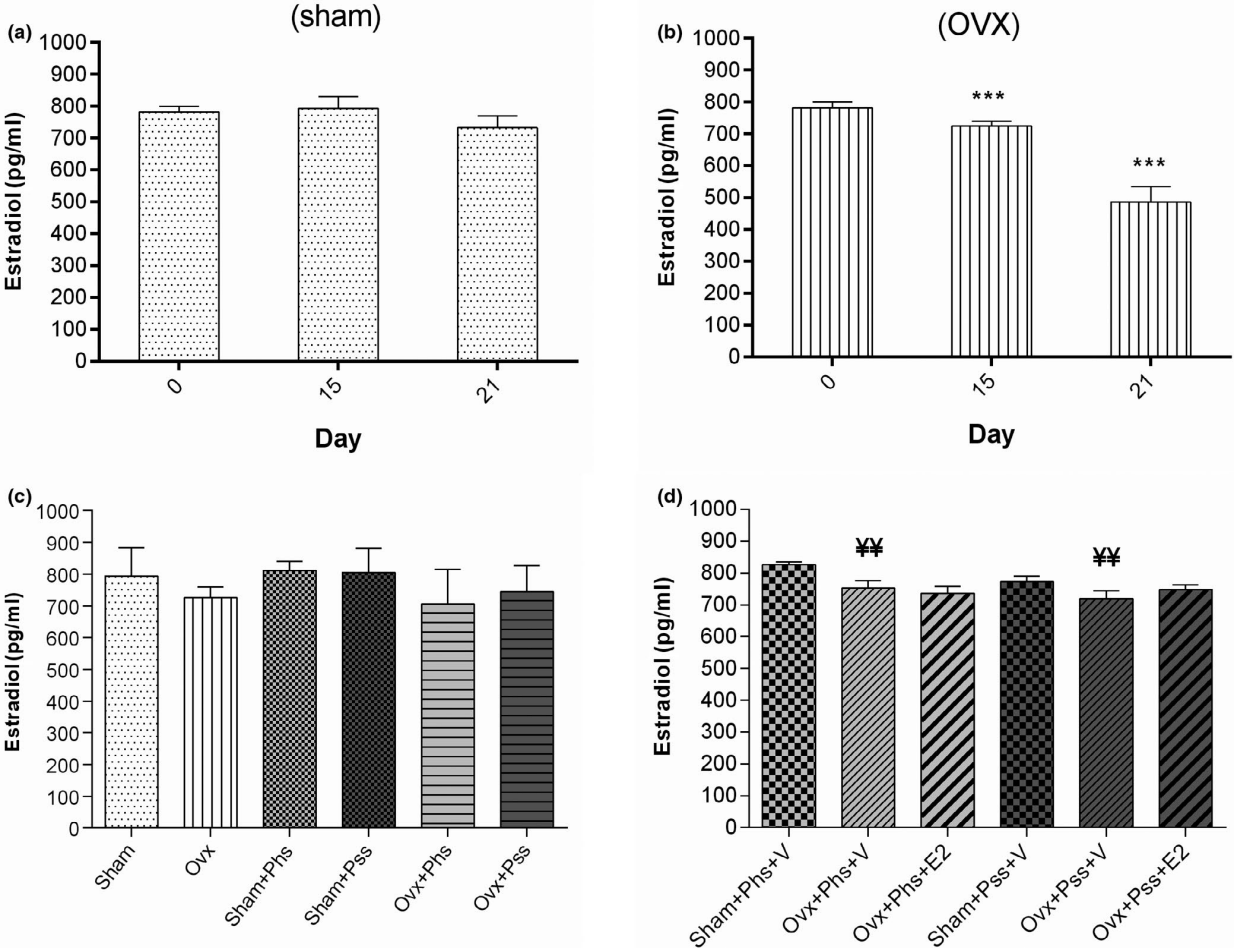
The effects of 17 β‐estradiol (E2), physical stress (Phs), and psychological stress (Pss) on E2 levels at 0, 15, and 21 days postsurgery (a). Ovariectomy (b and c) decreased E2 level on 15 and 21 days when compared to the level at 0 day. In addition, the E2 level on day 21 in OVX + Phs + V and OVX + Pss + V groups had significantly decreased when compared to sham + Phs + V and sham + Pss + V groups (d). One‐way ANOVA was used for analysis of these data. Mean ± *SEM*, (***) *p* < .001 versus Sham. (¥¥) *p* < .01 versus Sham + Phs + V and Sham + Pss + V

Within the OVX group, the plasma level of E2 was significantly decreased at 15 and 21 days postovariectomy when compared to levels detected on day 0 (Figure 6b, *p* < .001). Further, the plasma level of E2 level on day 21 in OVX rats exposed to the two stressors and vehicle was significantly lower when compared to the level of sham animals exposed to the stressors (Figure 6d, *p* < .01).

### 3.5 The effects of 17 β‐estradiol (E2), physical and psychological stress on plasma corticosterone levels on 0, 15 and 21 days

**FIGURE. 7:**
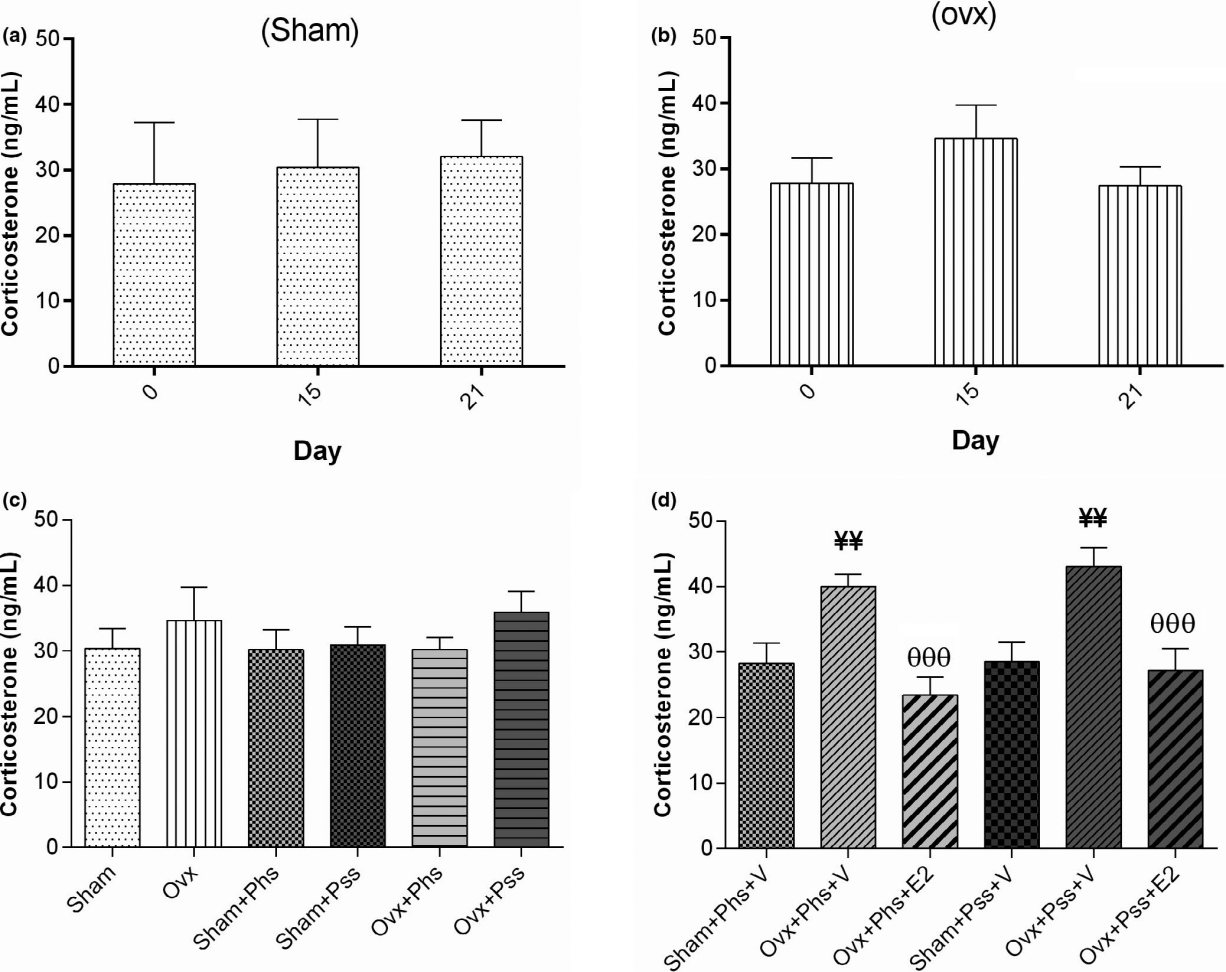
The effects of 17 β‐estradiol (E2), physical stress (Phs), and psychological stress (Pss) on corticosterone levels at 0, 15, and 21days following surgery (a, b and c). Ovariectomy in combination with stress increased corticostrone levels, whereas E2 decreased the levels (d). One‐way ANOVA was used for analysis of these data. Mean ± *SEM* (¥¥) *p* < .01 versus Sham + Phs + V and Sham + Pss + V. (θθθ) *p* < .001 versus OVX + Phs + V and OVX + Pss + V

The OVX groups exhibited no significant differences in blood corticosterone levels when comparing values at 0, 15, and 21 days postovariectomy indicating no effect of surgical menopause on corticosterone release (Figure 7a,b). Corticosterone levels in both OVX groups exposed to stressors were significantly higher than in the vehicle‐treated sham animals exposed to physical and psychological stress (Figure 7d, *p* < .01). Treatment of both OVX stressed groups with E2 resulted in significantly decreased blood corticosterone levels when compared to levels in vehicle‐treated stressed OVX animals (Figure 7d, *p* < .001).

## 4 DISCUSSION

In this study, the effectiveness of estrogen on stress‐based cognitive impairments including deficiencies in memory, exploratory behavior, and exhibition of anxiety‐like behaviors in female, ovariectomized rats was studied. Specifically, this research evaluated the impact of estrogen on physical or psychological stress‐induced learning and memory impairments, exploratory behavior, anxiety‐like behaviors, and corticosterone levels in a rat model of menopause. We confirmed that physical and psychological stress can impair cognition. Our findings revealed that physical and psychological stress disrupts spatial and passive avoidance learning of female rats. Further, physical and psychological stress alters exploratory and anxiety‐like behaviors as we saw an enhancement in anxiety, and a decrease in exploration in stressed, female rats. Results also revealed that physiological doses of estrogen in overiectimized rats improve the learning and memory impairments following physical and psychological stress.

While the role of stress in development of cognitive and anxiety‐like behavioral impairment is well established, the exact neurobiological mechanisms and possible interconnection of sex hormones are still unclear. While some level of stress hormones is crucial for appropriate functioning of the CNS, increases in their levels, especially if chronic, can result in a maladaptive response associated with cognitive dysfunctions. The ability of stress to alter cognitive‐based behaviors is likely due to the well‐known effects of stress hormones in altering neural function, and structure within neural groups controlling these behaviors. Stress has been reported to enhance anxiety and result in reductions in the size and shape of dendritic spines in the hippocampus, amygdala, and prefrontal cortex (Leuner & Shors, 2013). As dendritic spines receive the majority of excitatory synaptic contacts, these morphological changes would impact upon glutamatergic synapses, resulting in a reduction in the degree of excitatory neurotransmission (Leuner & Shors, 2013; Sigler et al., 2017). In the basolateral and central nuclei of the amygdala in female rats, reductions in levels of the protein spinophilin were induced by social isolation stress (Waddell et al., 2008). As spinophilin is a protein marker of dendritic spines (Allen et al., 1997), these data suggest that psychological stress induces reductions in spine density, and thus, excitatory transmission in this nucleus. While the mechanism(s) underlying the morphological changes is not fully understood, GR activation in the central nucleus of the amygdala (CeA) can result in anxiety‐type behaviors and an increased hormonal (CORT) response to a psychological stressor suggesting involvement of GCs in the structural and physiological changes (Weiser et al., 2010).

Our results showed that physical and psychological stress increased anxiety in female rats, and estrogen decreased this disorder in ovariectomized subjects, suggesting a role of estrogen in anxiety‐like behavior. In accordance with this suggestion, reductions in plasma levels of estradiol were accompanied by alterations in mood and enhanced levels of anxiety in healthy females 12 hr after parturition, and the relative levels of decrease in the estradiol level were positively correlated with the degree of anxiety reported (de Rezende et al., 2019). Previous animal studies have shown that ovariectomy induces anxiety, and estrogen administration in this surgical menopause model was able to reduce the negative response (Estrada et al., 2018). Estrogen was found to exert its decrease in anxiety‐like behavior through binding to estrogen beta receptors (ERβ) in the medial amygdala, which is critical in mediating emotionality (Estrada et al., 2018). Furthermore, modulation of the GR response by ERβ‐mediated signaling can be induced in this nucleus, as ERβ agonists reduced stress‐induced anxiety‐type behaviors and eliminated CORT secretion upon a stressor in the central nucleus of the amygdala (Weiser et al., 2010). In ovariectomized animals which exhibited higher levels of anxiety due to psychological stress compared with sham animals, estrogen attenuated release of CRF, and modulated HPA axis activity through ERβ in the paraventricular nucleus (Al‐Rahbi et al., 2013). One proposed mechanism for estrogen‐mediated reductions in stress‐induced anxiety in overiectamized rats is that ERβ signaling in amygdala reestablishes equilibrium between GABAergic and glutamatergic transmission in the basolateral amygdala through alterations in inhibitory and excitatory receptor up and downregulation (Tian et al., 2013).

Many studies have shown an effect of stress on spatial memory performance using the MWM test, albeit the majority of work has been conducted in males (Green & McCormick, 2013). In our study, we found that both physical stress and psychological stress had a detrimental impact on spatial memory when females were evaluated in the MWM. Further, E2 administration in ovariectomized subjects ameliorated the spatial memory deficit. While the mechanisms by which estrogen exerts its procognitive effects are not completely understood, estradiol, within the physiological range, is an important suppressor of the HPA stress response in females (Young et al., 2001). Previous investigations have reported that the hippocampus plays an important role in spatial memory formation in rodents and is negatively affected by effects of increased HPA axis activity (Kim et al., 2015).

High ACTH and corticosterone levels following HPA activity induced a loss of volume (Rahman et al., 2016), synaptic degeneration, neuronal atrophy (Popoli et al., 2012), decline in the number of dendrites, loss of dendritic spines, and deficits in neurogenesis (Conrad, 2008) in the hippocampus that resulted in spatial learning and memory impairments. While the molecular details underlying estrogen's effects on cognitive processes are not clear, ERβ and estrogen receptor alpha (ERα) immunoreactivity have been noted in the hippocampus within the dendrites of the principle cells, and it has been speculated that ERβ and ERα‐mediated nongenomic effects could play an important role in stabilizing glutamatergic hippocampal pyramidal cells which could underlie estrogen's protective effects on learning and memory (Milner et al., 2005). This conclusion is supported by findings that increasing levels of estrogen result in a diversity of changes in the hippocampus which would be promotive of learning and memory including augmentation of CA1 dendritic spine density (Woolley & McEwen, 1992), diminished long‐term depression (Vouimba et al., 2000) and enhanced long‐term potentiation (Warren et al., 1995). However, it should be noted that the duration of exposure to estrogen can have detrimental actions on hippocampal‐determined functioning, and its withdrawal followed by supplementation could be beneficial to cognitive processes, suggesting that the timing and kinetics of exposure to estrogen is a critical factor when considering its potential as a precognitive treatment via actions on hippocampal circuits. However, chronic remedy with estradiol ameliorates function on the radial arm maze (RAM; Luine et al., 1998), water maze (Daniel et al., 1999), and novel object recognition (Tuscher et al., 2015).

Both physical stress and psychological stress exerted a detrimental effect in PA testing, and estrogen was able to reduce the negative effects of both stressors and improve PA learning and memory in ovariectomized rats. Physical stress induced by chronic swimming has been shown to reduce PA memory, and this reduction in memory was attributed to an increase in stress‐induced endogenous opioids (Nazeri et al., 2016). However, in our present work, we saw that the application of physical and psychological stress to females without ovaries increased the level of corticosterone in the blood. Interestingly, ovarian resection did not increase blood corticosterone levels compared with the sham group in another study (Xu et al., 2015), which was in agreement with our work, indicating that surgery or ovarian resection has no effect on normal blood corticosterone levels in the absence of stress. Increases in levels of corticosterone in stressed animals have been seen following other studies using physical stress (Al‐Rahbi et al., 2014), and higher levels of corticosterone were shown to be importantly involved in the negative effect on the animal's subsequent learning and memory and effects were concentration‐dependent (Kovács et al., 1976), suggesting in ovariectomized female rats, corticosterone levels could play a role in learning and memory deficits. In our study, sham‐operated rats did not exhibit a rise in corticosterone upon stressor exposure, which is in contrast to findings that physical stress for two weeks increased blood corticosterone levels in ovarian intact animals, which could be due to differences in duration of the stress, the type of stress, the breed of the animal, or/and the age of the animal between the two studies (Al‐Rahbi et al., 2013).

Further, the behavioral effects on memory of stressors when assayed in the PA test depend on the severity, duration of the stress, and the stage of the animal within the sexual cycle. In male rats, long‐term restraint stress increased memory impairments as assayed in the PA test, which was an effect associated with decreases in hippocampal BDNF gene expression (Nooshinfar et al., 2011). Interestingly, BDNF expression in the hippocampus has been shown to be inhibited by corticosterone (Schaaf et al., 2000). Influences of physical stress on memory were found to differ across the estrus cycle in females, as periods with low estrogen levels (estrous) were associated with impaired memory in the PA test (Mohammadkhani et al., 2015). Interestingly, we found that not only the behavioral effects of physical and psychological stressors were on memory reduced by estrogen, but also the increased levels of corticosterone were significantly different in estrogen‐treated ovariectomized rats when compared to those treated with vehicle. When taken together with other studies, our data support the conclusion that the stress‐induced damaging effects on memory are mediated by corticosterone, putatively involving BDNF expression. Further, the low levels of estrogen in ovariectomized rats could play a role in the deficits as suggested by other studies (Al‐Rahbi et al., 2013), and, our data show that estrogen can protect against negative actions.

## 5 CONCLUSION

In general, results from the current research delineated that exposure to both physical stress and psychological stress can cause cognitive disorders and increase anxiety‐like behaviors in ovariectomized rats, which adds to the body of literature linking stress and mood disorders. Our results show that female rats are susceptible to both stressor types, and both stressors have an impact on motor and cognitive behaviors. Further, estrogen was able to reverse some of the behavioral deficits in ovariectomized females, which was an effect associated with reductions in stress‐induced corticosterone levels. Future investigations are required to determine the precise cellular and molecular pathways by which estrogen acts to reduce the behavioral deficits and steroid hormone levels to facilitate development of pharmacological interventions based on altering estrogen signaling in order to manage stress‐related disorders, especially in postmenopausal women.

## CONFLICT OF INTEREST

The authors declare no conflict of interest.

## AUTHOR CONTRIBUTION

MK conceived and designed the concept and road map of the study, searched the literature collected the data, and drafted the manuscript. MAR, HB, KAK, FM, MK, and MS critically reviewed the manuscript, designed the study, and helped in manuscript preparation. MK and MS are the archival authors and attest to the integrity of the original data and the analysis reported in this manuscript. All authors have made substantive contribution and attest to approving the final manuscript.

### PEER REVIEW

The peer review history for this article is available at https://publons.com/publon/10.1002/brb3.2139.

## Supporting information

Fig S1

Fig S2

## Data Availability

The data that support the findings of this study are available from the corresponding author upon reasonable request.
