# Supplementary figures and images for: Estrogen attenuates physical and psychological stress‐induced cognitive impairments in ovariectomized rats
Source: Brain Behav. 2021 Apr 3;11(5):e02139. doi: 10.1002/brb3.2139 (PMC8119870; doi:10.1002/brb3.2139)

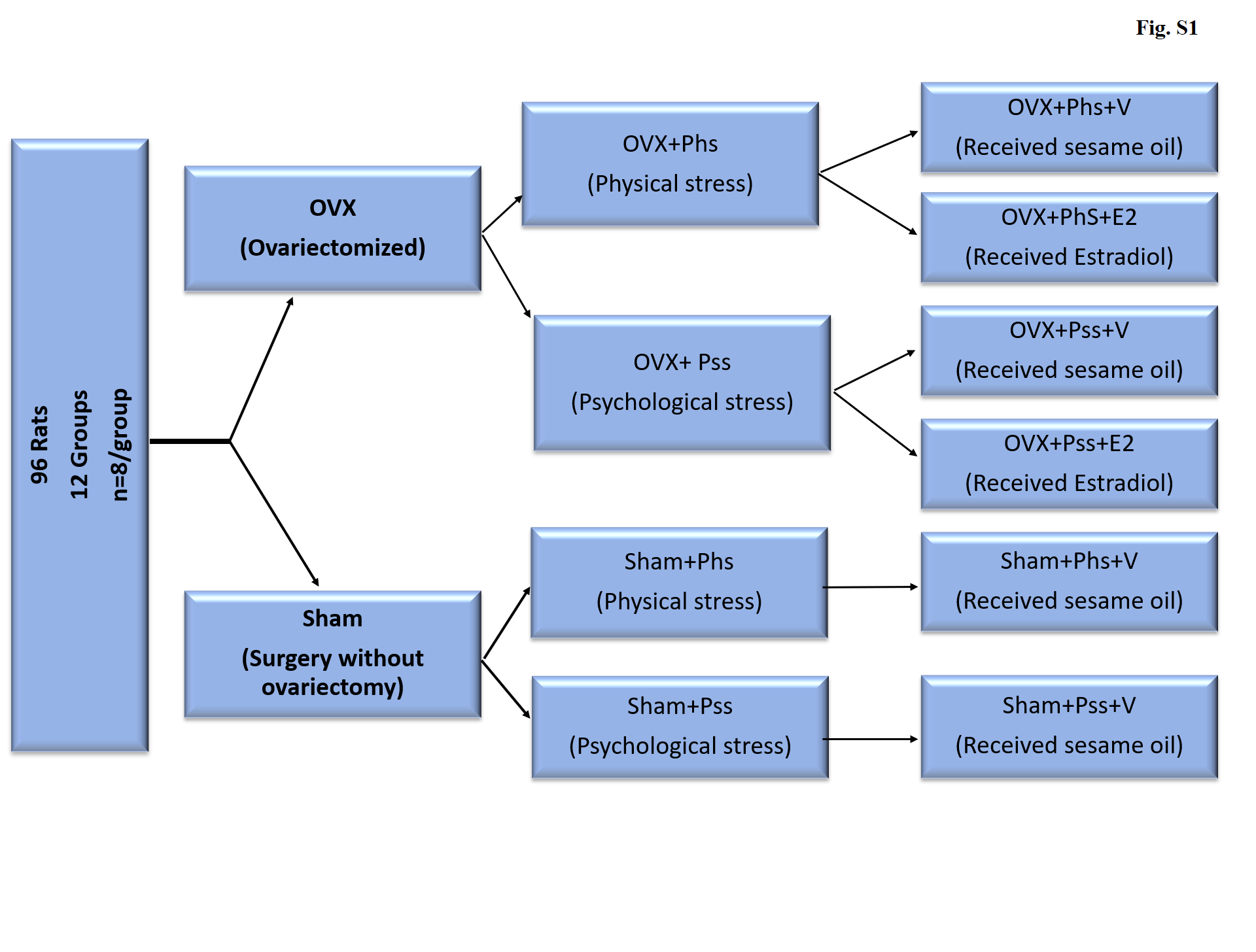

Supplement: Supplementary file 1 — Fig S1 [file BRB3-11-e02139-s001.tif]

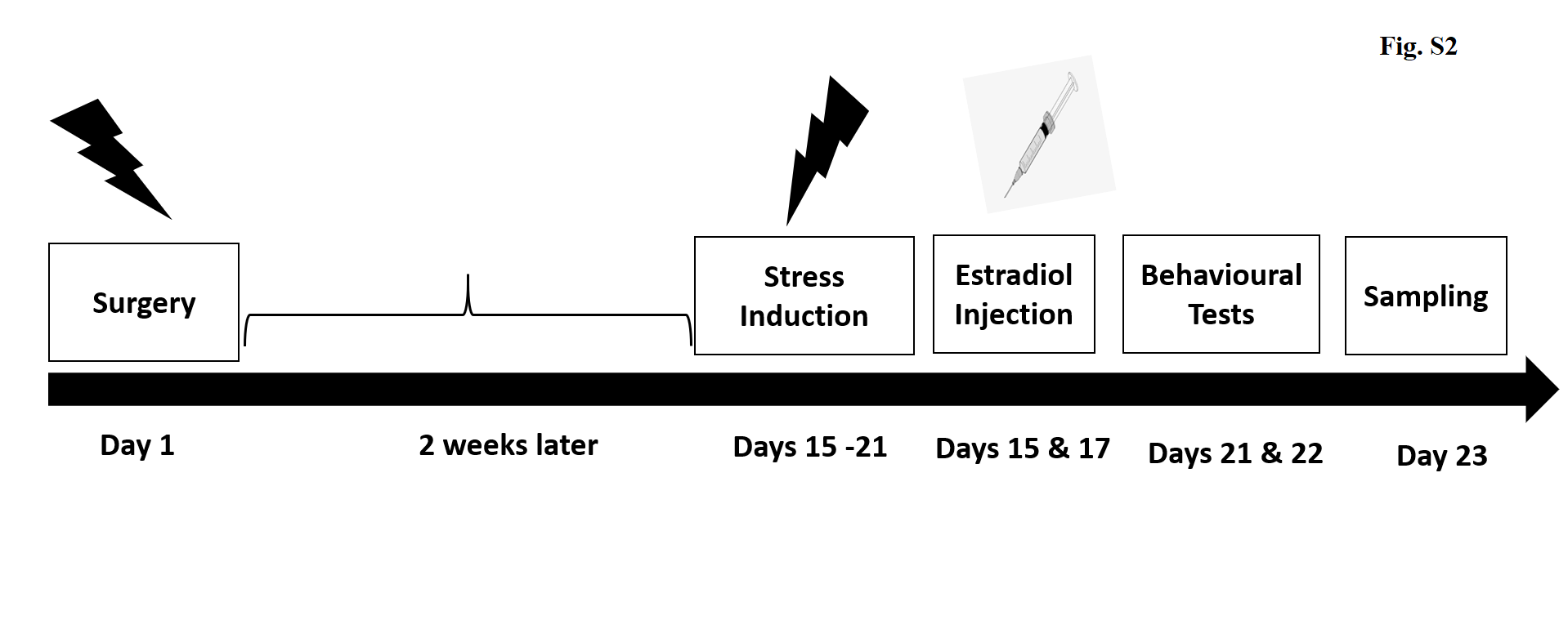

Supplement: Supplementary file 2 — Fig S2 [file BRB3-11-e02139-s002.tif]
